# Timing of physical activity in relation to liver fat content and insulin resistance

**DOI:** 10.1007/s00125-022-05813-3

**Published:** 2022-11-01

**Authors:** Jeroen H. P. M. van der Velde, Sebastiaan C. Boone, Esther Winters-van Eekelen, Matthijs K. C. Hesselink, Vera B. Schrauwen-Hinderling, Patrick Schrauwen, Hildo J. Lamb, Frits R. Rosendaal, Renée de Mutsert

**Affiliations:** 1 Department of Clinical Epidemiology, Leiden University Medical Center, Leiden, the Netherlands; 2 Department of Nutrition and Movement Sciences, Maastricht University Medical Center, Maastricht, the Netherlands; 3 Department of Radiology and Nuclear Medicine, Maastricht University Medical Center, Maastricht, the Netherlands; 4 Department of Radiology, Leiden University Medical Center, Leiden, the Netherlands

**Keywords:** Epidemiology, Insulin resistance, Liver fat, Physical activity, Sedentary behaviour, Sedentary breaks, Timing

## Abstract

**Aims/hypothesis:**

We hypothesised that the insulin-sensitising effect of physical activity depends on the timing of the activity. Here, we examined cross-sectional associations of breaks in sedentary time and timing of physical activity with liver fat content and insulin resistance in a Dutch cohort.

**Methods:**

In 775 participants of the Netherlands Epidemiology of Obesity (NEO) study, we assessed sedentary time, breaks in sedentary time and different intensities of physical activity using activity sensors, and liver fat content by magnetic resonance spectroscopy (*n*=256). Participants were categorised as being most active in the morning (06:00–12:00 hours), afternoon (12:00–18:00 hours) or evening (18:00–00:00 hours) or as engaging in moderate-to-vigorous-physical activity (MVPA) evenly distributed throughout the day. Most active in a certain time block was defined as spending the majority (%) of total daily MVPA in that block. We examined associations between sedentary time, breaks and timing of MVPA with liver fat content and HOMA-IR using linear regression analyses, adjusted for demographic and lifestyle factors including total body fat. Associations of timing of MVPA were additionally adjusted for total MVPA.

**Results:**

The participants (42% men) had a mean (SD) age of 56 (4) years and a mean (SD) BMI of 26.2 (4.1) kg/m^2^. Total sedentary time was not associated with liver fat content or insulin resistance, whereas the amount of breaks in sedentary time was associated with higher liver fat content. Total MVPA (−5%/h [95% CI −10%/h, 0%/h]) and timing of MVPA were associated with reduced insulin resistance but not with liver fat content. Compared with participants who had an even distribution of MVPA throughout the day, insulin resistance was similar (−3% [95% CI −25%, 16%]) in those most active in morning, whereas it was reduced in participants who were most active in the afternoon (−18% [95% CI −33%, −2%]) or evening (−25% [95% CI −49%, −4%]).

**Conclusions/interpretation:**

The number of daily breaks in sedentary time was not associated with lower liver fat content or reduced insulin resistance. Moderate-to-vigorous activity in the afternoon or evening was associated with a reduction of up to 25% in insulin resistance. Further studies should assess whether timing of physical activity is also important for the occurrence of type 2 diabetes.

**Graphical abstract:**

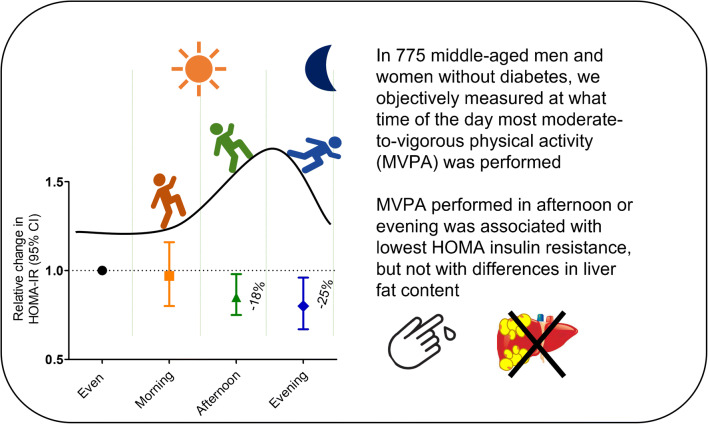

**Supplementary Information:**

The online version contains peer-reviewed but unedited supplementary material available at 10.1007/s00125-022-05813-3.

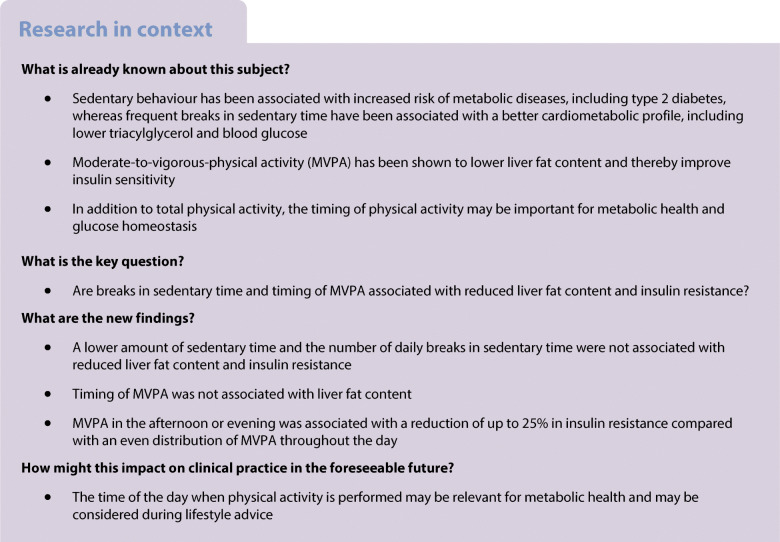



## Introduction

The current obesity pandemic is partially a consequence of lack of physical activity, and sedentary behaviour (prolonged sitting) during the day [1]. Sedentary behaviour is associated with an increased risk of cardiometabolic diseases, including type 2 diabetes [2–5]. Several studies have demonstrated that short breaks in sedentary time are associated with an improved cardiometabolic profile, including reduced triacylglycerol and glucose concentrations [6, 7]. Findings from observational studies are supported by (semi-)experimental studies showing that frequent interruptions of prolonged sitting with standing or light activities resulted in improved glycaemic responses [7–11] and triacylglycerol levels [9, 12]. High fasting serum triacylglycerol concentrations may reflect high liver fat content [13], which is strongly associated with insulin resistance [14]. Previous studies have shown that exercise is associated with reduced liver fat content and consequent improvement in insulin sensitivity [15]. Therefore, we hypothesised that breaks in sedentary time may reduce liver fat and insulin resistance, ultimately preventing type 2 diabetes.

Next to the duration of inactivity, it has been argued that the timing of physical activity during the day may be relevant for metabolic health [16, 17]. In animal models and in-vitro studies, differences in exercise capacity and associated metabolic pathways were observed in a daytime-dependent fashion [18–21]. Only a few studies have examined associations of timing of activities throughout the day with metabolic risk markers in humans, and results are inconsistent. For instance, a large observational study (*N*=7157) in women (mean age 71 years) reported that those who are less active during morning hours may be at higher risk of obesity [22]. This was, however, not observed in a smaller (*N*=125) study with a younger population (mean age 35 years) [23]. Further, high-intensity exercise in the afternoon improved blood glucose level more than high-intensity exercise in the morning in men with impaired glucose metabolism or diabetes [24, 25]. Recently, an association between morning bout-related moderate-to-vigorous-physical activity (MVPA) and increased cardiovascular risk in men with type 2 diabetes, compared with later timing of MVPA, has been reported [26]. The importance of timing of physical activity in relation to metabolic health in the general population is currently unknown.

Therefore, our aim was to investigate associations of timing of physical activity and breaks in sedentary time with liver fat content and insulin resistance in a middle-aged population.

## Methods

### Study design and population

The Netherlands Epidemiology of Obesity (NEO) study is a population-based, prospective cohort study designed to investigate pathways that lead to obesity-related diseases. Between 2008 and 2012, 6671 individuals aged 45–65 years were included, with an oversampling of individuals living with overweight or obesity. The study design and population is described in detail elsewhere [27]. Men and women living in the greater area of Leiden (in the west of the Netherlands) were invited to participate if they were aged between 45 and 65 years and had a self-reported BMI of 27 kg/m^2^ or higher. In addition, all inhabitants aged between 45 and 65 years from one municipality (Leiderdorp) were invited to participate, irrespective of their BMI. The Medical Ethical Committee of the Leiden University Medical Center (LUMC) approved the design of the study. All participants gave their written informed consent.

Participants came to the NEO study center of the LUMC for a study visit after an overnight fast of ≥10 h. Prior to this study visit, participants completed a questionnaire at home to report demographic, lifestyle and clinical information. All participants underwent a physical examination, including anthropometry and blood sampling, and completed a screening for potential contraindications for MRI. Approximately 35% of the participants without MRI contraindications were randomly selected to undergo assessment of liver fat content by proton magnetic resonance spectroscopy (^1^H-MRS). This study is a cross-sectional analysis of baseline measurements of the random subset of participants who carried an accelerometer.

### Data collection

#### Objective assessment of physical activity and sedentary time and breaks

Daily levels of activity were objectively assessed in a random subsample of NEO study participants (*n*=955) with a combined uniaxial acceleration and heart rate monitor (Actiheart; CamNtech, UK). Participants were instructed to wear the monitor continuously for four consecutive days and nights, and to carry on with all normal activities during this time.

Details of assessment have been described previously [28]. Using a branched equation algorithm, the acceleration and heart rate information (recorded in 15 s epochs) was summarised into estimates of physical activity energy expenditure (PAEE, in kJ kg^−1^ day^−1^) and time spent at different activity intensities was expressed as metabolic equivalents of task (MET) [29, 30].

Sedentary time was defined as time spent in activities with an intensity ≤1.5 MET, excluding sleep time, which was assumed as time between 23:00 hours and 07:30 hours on weekdays and between 23:30 hours and 08:30 hours on weekend days. A break in sedentary time was defined as a period of activity with an acceleration >0.75 m/s^2^ following a sedentary bout (uninterrupted period). Light physical activity (LPA) was defined as activity with an intensity >1.5 MET and ≤3 MET, and MVPA was defined as activity >3 MET. We differentiated the total amount of MVPA from MVPA accumulated in bouts ≥5 min.

Further, we divided the day into three 6 h blocks: 06:00–12:00 hours (morning); 12:00–18:00 hours (afternoon); and 18:00–24:00 hours (evening). For each time block we calculated the proportion of total daily MVPA that was spent in each 6 h time block. Based on these proportions, we categorised participants as most active in the morning, afternoon or evening, or as having an even distribution of MVPA throughout the day. For this, we used a minimum difference of 5% between time blocks. For example, an individual with 40%, 30% and 30% of total MVPA in the morning, afternoon and evening, respectively, was classified as most active in the morning, whereas a person with respective values of 35%, 33% and 32% was classified as having an even distribution of MVPA throughout the day. Participants were excluded from analyses if valid total wear time <24 h or if wear time within any single hour of the day was <30 min.

#### Blood sampling

Fasting blood samples were drawn from the antecubital vein after the participant had rested for 5 min in a seated position. Within 5 min after the first blood sample, participants drank a liquid mixed meal (total 400 ml, containing 2510 kJ (600 kcal)), with 16% of energy derived from protein, 50% from carbohydrates and 34% from fat. Two postprandial blood samples were drawn at 30 and 150 min after the mixed meal. Fasting and postprandial plasma glucose and serum insulin were determined as well as fasting HbA_1c_ concentrations [27]. We calculated the updated HOMA-IR from fasting glucose and insulin using the Oxford University online calculator (https://www.dtu.ox.ac.uk/homacalculator) and the Matsuda Insulin Sensitivity Index (Matsuda ISI).

#### Liver fat content

In a random subgroup of participants without MRI contraindications, liver fat content was quantified by ^1^H-MRS [31] on a 1.5 Tesla MR system (Philips Medical Systems, Best, the Netherlands). An 8 ml voxel was positioned in the right lobe of the liver, avoiding gross vascular structures and adipose tissue depots. Sixty-four averages were collected with water suppression. Spectra were obtained with an echo time of 26 ms and a repetition time of 3000 ms. Data points (1024) were collected using a 1000 Hz spectral line. Without changing any parameters, spectra without water suppression, with a repetition time of 10 s and with four averages, were obtained as an internal reference. ^1^H-MRS data were fitted using Java-based magnetic resonance user interface software (jMRUI version 2.2, Leuven, Belgium) [32]. Hepatic triacylglycerol content relative to water was calculated as the sum of signal amplitudes of methyl and methylene divided by the signal amplitude of water and then multiplied by 100. Non-alcoholic fatty liver disease (NAFLD) was defined as liver fat content ≥5.56% [33].

#### Covariates

On a questionnaire, participants reported ethnicity, which we grouped into White (reference category) and Other. Highest level of education was reported in ten categories according to the Dutch education system and grouped into high (including higher vocational school, and university) vs low education (reference). Tobacco smoking was reported in three categories: current; former; and never smoking (reference). Body weight and per cent body fat were estimated by the Tanita bio impedance balance (TBF-310; Tanita International Division, UK) with the participant not wearing shoes; 1 kg was subtracted from the body weight to account for clothing. BMI was calculated by dividing the body weight (kg) by the height squared (m^2^). Participants reported their habitual dietary intake (including alcohol intake) using a semi-quantitative self-administered 125-item food-frequency questionnaire [34, 35]. Dietary intake of nutrients and total energy was estimated using the Dutch Food Composition Table (NEVO-2011). We calculated the Dutch healthy diet index that indicated adherence to the Dutch Guidelines for a Healthy Diet of 2015 [36]. The context of physical activity (leisure or occupational) was derived from the Short Questionnaire to Assess Health-enhancing physical activity (SQUASH) [37].

### Statistical analyses

In the NEO study, individuals with a BMI of 27 kg/m^2^ or higher were oversampled. To correctly represent baseline associations in the general population, adjustments were made for this oversampling [38]. This was done by weighting all participants towards the BMI distribution of participants from the Leiderdorp municipality, the BMI distribution of whom was similar to the BMI distribution in the general Dutch population [39, 40]. Consequently, all results apply to a population-based study without oversampling of individuals with a high BMI.

Population characteristics were summarised as mean (SD), median (25th, 75th percentile) or proportion, stratified by timing of physical activity. As a consequence of the weighted analyses, no absolute numbers could be given, only proportions.

We examined associations between daily total sedentary time, the number of breaks in sedentary time and different intensities of physical activity (i.e. total PAEE, LPA, MVPA and MVPA in 5 min bouts) with liver fat content and insulin resistance using linear regression analyses.

For this, we constructed three models with potential confounding factors: model 1 was adjusted for age, sex, ethnicity and level of educational background; model 2 was additionally adjusted for lifestyle variables (alcohol consumption, smoking and Dutch healthy diet index); and model 3 was additionally adjusted for total body fat. As body fat may both confound and mediate the relationship between physical activity and liver fat content and insulin resistance, this model allows one to observe whether physical activity is associated with liver fat content and insulin resistance beyond effects via total body fat. In addition, associations with breaks in sedentary time were adjusted for total sedentary time and total volume of MVPA. Since liver fat content and insulin resistance were non-normally distributed, these were transformed using the natural logarithm. For interpretation of the results, the linear regression coefficients were back-transformed into a relative change (with 95% CIs). As an example, a relative change of 0.8 can be interpreted as 0.8-fold decrease in liver fat content for each hour of MVPA per day, reflecting a difference in liver fat content from, for example, 5% to 4%. Additionally, these relative changes were expressed as percentage change in the text: [exp(β)−1]×100 if β>0 and: −[1/exp(−β)−1]×100 if β<0, with 95% CI.

Next, using linear regression analyses, we examined liver fat content and insulin resistance in participants who were most active in morning, afternoon or evening compared with those with an even MVPA distribution. From the regression coefficients we calculated relative changes with 95% CIs. These analyses were adjusted for the same covariates as mentioned above as well as for total MVPA. To investigate whether associations were different between men and women, we tested for interaction by including product terms of sex and all physical activity variables in the adjusted models.

We repeated all analyses with timing of LPA and total PAEE. Further, we repeated all analyses for the following additional outcomes: insulin sensitivity; and fasting glucose, insulin and HbA_1c_. In addition, we added the amount of occupational activity and activity during leisure time as covariates to the models with timing of MVPA. Further, we restricted the analyses of breaks in sedentary behaviour to those with a similar amount of sedentary time (mean±1 SD).

Data was analysed using STATA 16.1 (StataCorp, College Station, TX, USA).

## Results

### Characteristics

**Fig. 1:**
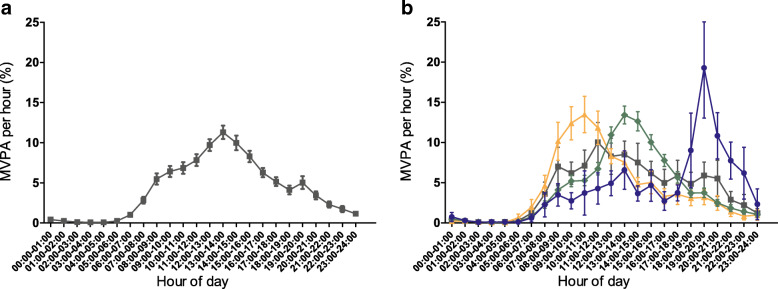
Distribution of MVPA presented as mean percentage with 95% CIs per hour throughout the day for total population (**a**) and for the four subgroups based on timing of MVPA (**b**). Orange triangles, most MVPA in the morning (16% of population); green diamonds, most MVPA in the afternoon (63% of population); blue squares, most MVPA in the evening (8% of population); grey squares, even distribution of MVPA throughout the day (13% of population). Results are based on analyses weighted towards the BMI distribution of the general population (*n*=775; i.e. 13% represents around 100 participants)

Physical activity was objectively assessed in 955 participants, of whom 932 had a successful measurement. After exclusion of participants with <24 h of total valid data or >30 min of missing data in any single hour (*n*=78), those working night shifts (*n*=2), those missing fasting blood samples (*n*=30), those using any type of glucose-lowering medication (*n*=35) and those missing covariates (*n*=12), data from 775 participants were used in the present analyses (see electronic supplementary material [ESM] Fig. 1), with a mean (SD) age of 56 (4) years and BMI of 26.2 (4.1) kg/m^2^; 42% were men. In the total study population, median liver fat content was 2.6% (IQR 1.1–5.8) with 28% NAFLD, and median HOMA-IR was 1.0 (IQR 0.7–1.5). Characteristics of the total study population and of participants stratified by the time of day when most of total daily MVPA was performed are shown in Table 1.

**Table 1:** Characteristics of the total study population and stratified by the time of day during which most of the total daily MVPA is performed

Characteristic	Total population	Even distribution of MVPA (13%)	Most MVPA in morning (16%)	Most MVPA in afternoon (63%)	Most MVPA in evening (8%)
Sex, % women	58	52	56	59	63
Age, years	56 (6)	56 (7)	55 (6)	56 (6)	55 (7)
Education, % high	40	38	39	39	54
Ethnicity, % White	96.0	94.3	93.4	96.4	100
BMI, kg/m^2^	26.2 (4.1)	27.0 (3.8)	26.0 (4.1)	26.2 (4.2)	26.6 (4.0)
Smoking, % current	15	17	16	14	21
Dutch healthy diet index	71 (14)	71 (15)	70 (15)	71 (14)	70 (16)
CVD history, %	5	4	2	7	2
Total Actiheart wear time, h	84.7 (11.3)	84.9 (13.3)	83.5 (10.7)	85.0 (10.5)	84.1 (14.9)
Total PAEE, kJ kg^−1^ day^−1^	45.9 (18.0)	46.7 (21.4)	49.2 (18.7)	45.6 (17.7)	39.4 (12.6)
Sedentary time, h/day	9.1 (2.1)	8.9 (2.4)	8.9 (2.1)	9.1 (2.1)	10.0 (1.8)
Breaks in sedentary time, *n*/day	71 (16)	70 (18)	70 (16)	72 (16)	68 (17)
LPA, h/day	5.4 (1.7)	5.2 (1.8)	5.4 (1.7)	5.4 (1.7)	4.9 (1.6)
MVPA, h/day	1.2 (0.7–1.8)	1.1 (0.8–1.9)	1.4 (0.7–2.2)	1.2 (0.7–1.7)	1.2 (0.7–1.5)
MVPA in 5 min bouts, h/day	0.5 (0.2–0.9)	0.5 (0.2–0.8)	0.7 (0.2–1.3)	0.5 (0.2–0.9)	0.5 (0.2–0.7)
Vigorous physical activity, min/day	1.2 (0–7.6)	0.1 (0–4.6)	2.1 (0–8.5)	1.3 (0–7.6)	2.0 (0.1–12.3)
Leisure-time physical activity, MET-h/week	29.6 (17.0–48.0)	24.3 (14.0–52.0)	31.5 (15.3–49.8)	30.7 (19.0–45.0)	34.0 (16.0–51.5)
Liver fat content, %	2.6 (1.1–5.8)	3.4 (1.9–4.9)	2.5 (1.1–5.1)	2.2 (1.1–5.8)	3.6 (0.9–6.3)
NAFLD, % liver fat ≥5.56%	28	24	21	26	39
HOMA-IR	1.0 (0.7–1.5)	1.3 (1.0–1.7)	1.0 (0.6–1.7)	1.0 (0.7–1.4)	0.8 (0.7–1.4)
Matsuda ISI	5.6 (3.8–8.2)	4.9 (3.7–6.5)	5.2 (4.0–8.1)	5.6 (3.8–8.6)	6.1 (3.9–8.3)

Results are presented as mean (SD), median (25th–75th percentile) or percentage

Results are based on analyses weighted towards the BMI distribution of the general population (*n*=775; or *n*=206 in those with liver fat content)

### Associations of sedentary time, breaks in sedentary time and different intensities of physical activity with liver fat content and insulin resistance

Table 2 presents the associations of sedentary time, breaks from sedentary time and physical activity with liver fat content and insulin resistance. Neither sedentary time nor breaks in sedentary time were associated with lower insulin resistance. However, the number of breaks in sedentary time was associated with 22% (95% CI 9%, 37%) higher liver fat content. This association remained when analyses were stratified by daily sedentary time (data not shown). Total PAEE, MVPA and MVPA in 5 min bouts were associated with liver fat content in models 1 and 2 but associations were attenuated after adjustment for total body fat. Similarly, total PAEE, MVPA and MVPA in 5 min bouts, but not LPA, were associated with insulin resistance in models 1 and 2. After adjustment for total body fat, an additional hour of MVPA was associated with −5% (95% CI −10%, 0%) insulin resistance and an additional hour of MVPA in 5 min bouts was associated with −9% (95% CI −16%, −2%) insulin resistance. Even though the amount of liver fat was higher in men (median 4.1%) than in women (median 1.6%), we did not observe an interaction effect of sex in any of the associations (all *p*>0.10).

**Table 2:** Relative changes in liver fat content and insulin resistance according to sedentary time, number of breaks in sedentary time and different levels of physical activity intensity

Physical activity	Relative change (95% CI)	
	Liver fat content^a^	Insulin resistance (HOMA-IR)^b^
Sedentary time, h/day		
Model 1^c^	1.04 (0.96, 1.12)	1.02 (0.99, 1.04)
Model 2^d^	1.06 (0.98, 1.13)	1.02 (0.99, 1.04)
Model 3^e^	1.01 (0.95, 1.07)	1.01 (0.98, 1.03)
Breaks in sedentary time, 10/day		
Model 1^c^	1.09 (0.97, 1.21)	0.97 (0.93, 1.01)
Model 2^d^	1.09 (0.99, 1.21)	0.97 (0.93, 1.01)
Model 3^e^	1.13 (1.05, 1.23)	0.98 (0.95, 1.02)
Model 3 + ST and MVPA	1.22 (1.09, 1.37)	0.98 (0.94, 1.02)
Total PAEE, 10 kJ kg^−1^ day^−1^		
Model 1^c^	0.90 (0.83, 0.99)	0.95 (0.93, 0.98)
Model 2^d^	0.89 (0.82, 0.97)	0.96 (0.93, 0.99)
Model 3^e^	0.98 (0.91, 1.07)	0.98 (0.95, 1.00)
LPA, h/day		
Model 1^c^	0.98 (0.88, 1.08)	0.99 (0.96, 1.02)
Model 2^d^	0.96 (0.88, 1.06)	0.99 (0.96, 1.02)
Model 3^e^	1.01 (0.93, 1.10)	1.00 (0.97, 1.03)
MVPA, h/day		
Model 1^c^	0.80 (0.66, 0.97)	0.92 (0.86, 0.97)
Model 2^d^	0.77 (0.64, 0.94)	0.92 (0.87, 0.97)
Model 3^e^	0.97 (0.80, 1.18)	0.95 (0.90, 1.00)
MVPA in 5 min bouts, h/day		
Model 1^c^	0.68 (0.51, 0.92)	0.87 (0.81, 0.93)
Model 2^d^	0.66 (0.50, 0.89)	0.87 (0.81, 0.94)
Model 3^e^	0.94 (0.70, 1.26)	0.91 (0.86, 0.98)

Results are based on analyses weighted towards the BMI distribution of the general population and were derived from regression coefficients with 95% CIs from linear regression analyses and expressed as a relative change. Such relative change can be interpreted as a ratio (e.g. 0.8, can be interpreted as 0.8-fold reduced liver fat content for each hour of MVPA per day, which would reflect a decrease in liver fat content from, for example, 5% to 4%). Further, these relative changes were expressed as percentage change in the text: [exp(β)−1]×100 if β>0 and: −[1/exp(−β)−1]×100 if β<0, with 95% CI , e.g. a relative change of 0.80 (0.67, 0.96) corresponds with −25% (−49%, −4%) percentage change

^a^Participants with alcohol consumption ≥40 g per day were excluded from the analyses

^b^Associations with insulin resistance were based on the total study population (*n*=775), and those with liver fat content (*n*=206) measured by ^1^H-MRS

^c^Model 1: associations were adjusted for age, sex, educational background and ethnicity

^d^Model 2: as for model 1, additionally adjusted for alcohol consumption, smoking and the Dutch healthy diet index

^e^Model 3: as for model 2, additionally adjusted for total body fat

ST, sedentary time

ESM Table 1 shows relative changes in insulin sensitivity, fasting concentrations of glucose, insulin and HbA_1c_ for sedentary time, number of breaks in sedentary time and different levels of physical activity intensity. Associations with the Matsuda ISI showed similar patterns to associations with insulin resistance. Overall, we observed associations with fasting insulin rather than with fasting glucose or HbA_1c_.

### Association of timing of physical activity with liver fat content and insulin resistance

**Fig. 2:**
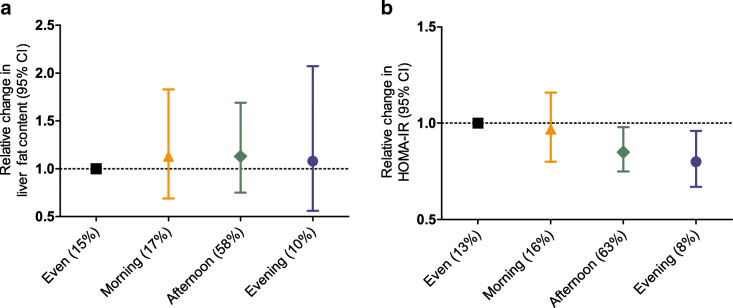
Relative changes in liver fat content (**a**) and insulin resistance (**b**) between subgroups based on timing of MVPA, compared with an even distribution of MVPA throughout the day. All associations were adjusted for age, sex, educational background, ethnicity, alcohol consumption, smoking, Dutch healthy diet index, total body fat and total MVPA. Results are based on analyses weighted towards the BMI distribution of the general population and were derived from regression coefficients with 95% CIs from linear regression analyses and expressed as a relative change. Such relative change can be interpreted as a ratio (e.g. 0.8 can be interpreted as 0.8-fold reduced liver fat content for most MVPA in the evening vs an even distribution of MVAP, which would reflect a decrease in liver fat content from, for example, 5% to 4%). Associations with insulin resistance were based on the total study population (*n*=775; i.e. 13% represents around 100 participants). Associations with liver fat content were based on participants with ^1^H-MRS of liver fat (*n*=206; i.e. 15% represents around 31 participants). Percentages in brackets indicate proportion of the study population within the subgroups with most MVPA in the morning, afternoon or evening, or with an even distribution of MVPA throughout the day. Participants with alcohol consumption ≥40 g per day were excluded from the analyses of liver fat content

Figure 1 illustrates the distribution of MVPA throughout the day for the total population and for the four subgroups based on timing of MVPA. Crude differences in liver fat content and insulin resistance between subgroups based on timing of MVPA are shown in ESM Fig. 2. After adjustments, insulin resistance was reduced in participants who were most active in the afternoon (−18% [95% CI −33%, −2%]) or evening (−25% [95% CI −49%, −4%]), whereas insulin resistance was unaffected in those most active in the morning (−3% [95% CI −25%, 16%]) compared with participants with an even distribution of MVPA (Fig. 2). Similar observations were made upon studying subgroups in which MVPA was assessed in 5 min bouts (ESM Table 2). Timing of MVPA was not associated with liver fat content. Additional adjustment for the amount of occupational and leisure-time physical activity did not affect the observed associations (not shown). ESM Table 2 shows the associations between timing of MVPA and additional outcomes. Here, we also observed associations with fasting insulin rather than with fasting glucose: insulin was reduced in participants who did most of their MVPA in the afternoon (−18% [95% CI −35%, −2%]) or in the evening (−27% [95% CI −52%, −5%]).

There were no marked differences in liver fat content and insulin resistance between groups based on timing of LPA. Participants who had their peak total PAEE in the morning, afternoon or evening were less insulin resistant than those who had an even distribution of PAEE throughout the day (ESM Table 3).

## Discussion

In our study of middle-aged men and women, we did not observe associations of sedentary time with liver fat or insulin resistance, nor did we observe associations of number of breaks in sitting time with reduced liver fat or insulin resistance. However, higher total PAEE and, particularly, more MVPA was associated with reduced liver fat and insulin resistance. Interestingly, we observed that the timing of MVPA during the day was associated with insulin resistance: MVPA in the afternoon or evening but not in the morning was associated with reduced insulin resistance compared with having an even distribution of MVPA during the day.

Previous observational and experimental studies have reported positive effects of breaks in sitting time on different metabolic outcomes, such as reduced body weight, triacylglycerol concentrations and insulin resistance [7–10]. However, in our study, the number of breaks was not associated with reduced insulin resistance. One explanation could be that experimental studies usually examine regular breaks in sedentary time vs continuous sitting [9]. This contrast between an almost complete absence of breaks in sedentary time and a situation with regular breaks is not common in a free-living setting, such as in our study, where we observed an average of 71 breaks in combination with 9 h sedentary time. Another explanation may be that in our study the intensity of the activity during the breaks was too light to elicit metabolic responses. Most daily activities are of light intensity and because we did not observe an association between LPA and insulin resistance, this may also explain the lack of an association between breaks and insulin resistance. Similarly, in a previous study we observed that MVPA but not LPA was associated with less total body fat, visceral fat and liver fat [28].

In contrast to our hypothesis, we observed that a greater number of breaks in sedentary time was associated with somewhat more liver fat instead of less liver fat. We reasoned that total sedentary time might explain this result, as people who sit more also have more opportunity to break their sitting time. However, we still observed a positive association between the number of breaks and liver fat content within those with similar (mean±1SD; 7.1–11.3 h/day) sedentary time. Because the number of breaks was not similarly related to increased insulin resistance and the range of liver fat content in our population was as could be expected [33], we cannot think of any explanation other than chance as a result of the small sample size in which these analyses were performed (*n*=206). Overall, there is still much debate as to whether breaks in sedentary time per se have protective cardiometabolic effects [41] and longer intervention trials are needed to show long-term health benefits of short, light-intensity breaks in sitting time.

Timing of exercise is a relatively unexplored field in human studies. We observed that MVPA in the afternoon or evening, compared with an even distribution of MVPA during the day, was associated with reduced insulin resistance. Our findings are supported by trials that have shown that in men who were metabolically compromised [24] and in men with type 2 diabetes [25] high-intensity exercise in the afternoon was more efficacious in improving blood glucose than high-intensity exercise in the morning. However, another trial in a similar population did not show this [42]. Further, a trial in treated hypertensive men showed that aerobic training performed in the evening but not in the morning decreased clinically determined and ambulatory BP [43]. To date, one other observational study involving objectively assessed physical activity has examined the role of timing of MVPA in cardiometabolic health. Using data from The Look AHEAD trial, researchers reported an association between morning bout-related MVPA and increased risk of CHD in men compared with MVPA later in the day [26].

The mechanisms underlying the potential benefits of timing of MVPA remain unclear. Because timing of MVPA and liver fat content were not associated in our study, reduced liver fat accumulation is unlikely to underlie the relationship between MVPA in the afternoon or evening and reduced insulin resistance. Since physical activity may act as a Zeitgeber, a cue for the activation of clock genes, it has been argued that timing of physical activity may enhance the circadian rhythm and consequently metabolic health [16]. Indeed, mechanistic studies have previously shown that metabolic responses to MVPA differed based on the time of the day when MVPA was performed, and that these metabolic responses were regulated by the clock genes [18, 19]. Furthermore, it was shown that muscular strength and skeletal muscle mitochondrial function peak in the late afternoon, suggesting a circadian rhythm for oxidative metabolism [44]. Thus, being most active during this period may elicit greater metabolic responses than earlier in the day.

In our study, only timing of MVPA, and not timing of LPA or total PAEE, was associated with insulin resistance. This is in line with observations in mice where no difference in insulin signalling was observed when only light-intensity activity was performed at different time points [20]. Thus, the effects of timing of physical activity may only result from high-intensity activity. Further, in our study, the finding that most MVPA in the afternoon and evening was associated with reduced insulin resistance mainly resulted from lower fasting insulin rather than differences in fasting glucose concentrations. These findings provide some insight for future studies that further examine the underlying mechanisms behind the potential benefits of timing of physical activity.

### Strengths and limitations

Strengths of our study include the objective assessment of physical activity with an accelerometer combined with heart rate monitor, the population-based setting and the adjustment for important confounding factors.

However, several limitations of our study should be considered. First, inherent to the study’s observational design, residual confounding may still be present. For example, the time of day at which people engage in most MVPA may depend on the context of the physical activity. One could argue that MVPA later in the day is mainly performed during leisure time. Leisure-time physical activity has been associated with improved metabolic health, whereas occupational physical activity has sometimes even been associated with adverse health effects [45]. However, adding occupational or leisure-time physical activity to the regression models did not change our findings. Second, the Actiheart is a chest-worn accelerometer and is therefore less valid for estimating sedentary time and breaks than thigh-worn accelerometers. This may have led to an over- or underestimation of sedentary time or breaks in sedentary time in some participants. Although such misclassification would not be related to the outcomes of our study, this may have resulted in dilution of the associations of sedentary time and breaks. In addition, the objective assessment of physical activity was limited to 4 days and yielded limited data for weekend days. Activity patterns may vary from day to day and between week and weekend days. Consequently, by summarising data into an average 24 h period, these variations are not considered and misclassification of timing of MVPA may have occurred. However, we expect this would have led to an underestimation of the observed associations with reduced insulin resistance in people who were active in the afternoon or evening. Finally, we lacked information on the participants’ chronotype and the effects of timing of physical activity may depend on an individual’s chronotype [46]. Since a late chronotype has been associated with increased risks of type 2 diabetes and the metabolic syndrome [47], it may be important to examine health effects of timing of MVPA relative to each person’s individual chronotype.

### Conclusion

In conclusion, in contrast to our hypothesis, a lower amount of sedentary time or more breaks in sedentary time were not associated with reduced liver fat content or insulin resistance. However, in addition to the total amount of daily MVPA, timing of MVPA during the day was associated with reduced insulin resistance: performing most MVPA in the afternoon or evening was associated with up to 25% reduced insulin resistance compared with an even distribution of MVPA during the day. These results suggest that timing of physical activity throughout the day is relevant for the beneficial effects of physical activity on inulin sensitivity. Further studies should assess whether timing of physical activity is indeed important for the occurrence of type 2 diabetes, taking into account the influence of chronotype.

## Supplementary Information

ESM (PDF 491 kb)

## Abbreviations

^1^H-MRS: Proton magnetic resonance spectroscopy
ISI: Insulin sensitivity index
LPA: Light physical activity
LUMC: Leiden University Medical Center
MET: Metabolic equivalents of task
MVPA: Moderate-to-vigorous-physical activity
NAFLD: Non-alcoholic fatty liver disease
NEO: Netherlands Epidemiology of Obesity
PAEE: Physical activity energy expenditure

## References

[1] WHO (2022) WHO European Regional Obesity Report 2022. Copenhagen: WHO Regional Office for Europe. Licence: CC BY-NC-SA 3.0 IGO

[2] Brocklebank LA, Falconer CL, Page AS, Perry R, Cooper AR (2015). Accelerometer-measured sedentary time and cardiometabolic biomarkers: A systematic review. Prev Med.

[3] Biswas A, Oh PI, Faulkner GE (2015). Sedentary time and its association with risk for disease incidence, mortality, and hospitalization in adults: a systematic review and meta-analysis. Ann Intern Med.

[4] van der Berg JD, Stehouwer CD, Bosma H (2016). Associations of total amount and patterns of sedentary behaviour with type 2 diabetes and the metabolic syndrome: The Maastricht Study. Diabetologia.

[5] Patterson R, McNamara E, Tainio M (2018). Sedentary behaviour and risk of all-cause, cardiovascular and cancer mortality, and incident type 2 diabetes: a systematic review and dose response meta-analysis. Eur J Epidemiol.

[6] Healy GN, Dunstan DW, Salmon J (2008). Breaks in sedentary time beneficial associations with metabolic risk. Diabetes Care.

[7] Chastin SF, Egerton T, Leask C, Stamatakis E (2015). Meta-analysis of the relationship between breaks in sedentary behavior and cardiometabolic health. Obesity.

[8] Duvivier BM, Schaper NC, Hesselink MK (2017). Breaking sitting with light activities vs structured exercise: a randomised crossover study demonstrating benefits for glycaemic control and insulin sensitivity in type 2 diabetes. Diabetologia.

[9] Loh R, Stamatakis E, Folkerts D, Allgrove JE, Moir HJ (2020). Effects of interrupting prolonged sitting with physical activity breaks on blood glucose, insulin and triacylglycerol measures: a systematic review and meta-analysis. Sports Med.

[10] Saunders TJ, Atkinson HF, Burr J, MacEwen B, Skeaff CM, Peddie MC (2018). The acute metabolic and vascular impact of interrupting prolonged sitting: a systematic review and meta-analysis. Sports Med.

[11] Remie CM, Janssens GE, Bilet L (2021). Sitting less elicits metabolic responses similar to exercise and enhances insulin sensitivity in postmenopausal women. Diabetologia.

[12] Duvivier BM, Schaper NC, Koster A (2017). Benefits of substituting sitting with standing and walking in free-living conditions for cardiometabolic risk markers, cognition and mood in overweight adults. Front Physiol.

[13] Hazlehurst JM, Woods C, Marjot T, Cobbold JF, Tomlinson JW (2016). Non-alcoholic fatty liver disease and diabetes. Metabolism.

[14] Bays H, Mandarino L, DeFronzo RA (2004). Role of the adipocyte, free fatty acids, and ectopic fat in pathogenesis of type 2 diabetes mellitus: peroxisomal proliferator-activated receptor agonists provide a rational therapeutic approach. J Clin Endocrinol Metab.

[15] Brouwers B, Hesselink MK, Schrauwen P, Schrauwen-Hinderling VB (2016). Effects of exercise training on intrahepatic lipid content in humans. Diabetologia.

[16] Qian J, Scheer FA (2016). Circadian system and glucose metabolism: implications for physiology and disease. Trends Endocrinol Metab.

[17] Stenvers DJ, Scheer FA, Schrauwen P, la Fleur SE, Kalsbeek A (2019). Circadian clocks and insulin resistance. Nat Rev Endocrinol.

[18] Ezagouri S, Zwighaft Z, Sobel J et al (2019) Physiological and molecular dissection of daily variance in exercise capacity. Cell Metab 30:78–91. e74. 10.1016/j.cmet.2019.03.012 10.1016/j.cmet.2019.03.012 31006590

[19] Sato S, Basse AL, Schönke M et al (2019) Time of exercise specifies the impact on muscle metabolic pathways and systemic energy homeostasis. Cell Metab 30:92–110. e114. 10.1016/j.cmet.2019.03.013 10.1016/j.cmet.2019.03.013 31006592

[20] Dalbram E, Basse AL, Zierath JR, Treebak JT (2019). Voluntary wheel running in the late dark phase ameliorates diet-induced obesity in mice without altering insulin action. J Appl Physiol.

[21] Sato S, Dyar KA, Treebak JT et al (2022) Atlas of exercise metabolism reveals time-dependent signatures of metabolic homeostasis. Cell Metab 34:329–345 e328. 10.1016/j.cmet.2021.12.016 10.1016/j.cmet.2021.12.016 PMC13189211 35030324

[22] Chomistek AK, Shiroma EJ, Lee I-M (2016). The relationship between time of day of physical activity and obesity in older women. J Phys Act Health.

[23] Marinac CR, Quante M, Mariani S (2019). Associations between timing of meals, physical activity, light exposure, and sleep with body mass index in free-living adults. J Phys Act Health.

[24] Mancilla R, Brouwers B, Schrauwen-Hinderling VB, Hesselink MK, Hoeks J, Schrauwen P (2021). Exercise training elicits superior metabolic effects when performed in the afternoon compared to morning in metabolically compromised humans. Physiol Rep.

[25] Savikj M, Gabriel BM, Alm PS (2019). Afternoon exercise is more efficacious than morning exercise at improving blood glucose levels in individuals with type 2 diabetes: a randomised crossover trial. Diabetologia.

[26] Qian J, Walkup MP, Chen S-H (2021). Association of objectively measured timing of physical activity bouts with cardiovascular health in type 2 diabetes. Diabetes Care.

[27] de Mutsert R, Den Heijer M, Rabelink TJ (2013). The Netherlands Epidemiology of Obesity (NEO) study: study design and data collection. Eur J Epidemiol.

[28] Winters-van Eekelen E, van der Velde JH, Boone SC (2021). Objectively measured physical activity and body fatness: associations with total body fat, visceral fat, and liver fat. Med Sci Sports Exerc.

[29] Brage S, Brage N, Franks PW (2004). Branched equation modeling of simultaneous accelerometry and heart rate monitoring improves estimate of directly measured physical activity energy expenditure. J Appl Physiol.

[30] Brage S, Brage N, Franks P, Ekelund U, Wareham N (2005). Reliability and validity of the combined heart rate and movement sensor Actiheart. Eur J Clin Nutr.

[31] Van Der Meer RW, Hammer S, Lamb HJ (2008). Effects of short-term high-fat, high-energy diet on hepatic and myocardial triglyceride content in healthy men. J Clin Endocrinol Metab.

[32] Naressi A, Couturier C, Devos J (2001). Java-based graphical user interface for the MRUI quantitation package. Magn Reson Mater Phys Biol Med.

[33] Szczepaniak LS, Nurenberg P, Leonard D (2005). Magnetic resonance spectroscopy to measure hepatic triglyceride content: prevalence of hepatic steatosis in the general population. Am J Physiol Endocrinol Metab.

[34] Siebelink E, Geelen A, de Vries JH (2011). Self-reported energy intake by FFQ compared with actual energy intake to maintain body weight in 516 adults. Br J Nutr.

[35] Verkleij-Hagoort AC, de Vries JH, Stegers MP, Lindemans J, Ursem NT, Steegers-Theunissen RP (2007). Validation of the assessment of folate and vitamin B12 intake in women of reproductive age: the method of triads. Eur J Clin Nutr.

[36] Looman M, Feskens EJ, de Rijk M (2017). Development and evaluation of the Dutch Healthy Diet index 2015. Public Health Nutr.

[37] Wendel-Vos GW, Schuit AJ, Saris WH, Kromhout D (2003). Reproducibility and relative validity of the short questionnaire to assess health-enhancing physical activity. J Clin Epidemiol.

[38] Korn EL, Graubard BI (1991). Epidemiologic studies utilizing surveys: accounting for the sampling design. Am J Public Health.

[39] Lumley T (2004). Analysis of complex survey samples. J Stat Softw.

[40] Ministerie van Ministerie van Volksgezondheid, Welzijn en Sport (Dutch Ministry of Health, Welfare and Sport). "Overgewicht" Internet: https://www.volksgezondheidenzorg.info/onderwerp/overgewicht/cijfers-context/huidige-situatie (accessed February 20 2017)

[41] Stamatakis E, Ekelund U, Ding D, Hamer M, Bauman AE, Lee I-M (2019). Is the time right for quantitative public health guidelines on sitting? A narrative review of sedentary behaviour research paradigms and findings. Br J Sports Med.

[42] Teo SY, Kanaley JA, Guelfi KJ, Marston KJ, Fairchild TJ (2020). The impact of exercise timing on glycemic control: a randomized clinical trial. Med Sci Sports Exerc.

[43] Brito L, Pecanha T, Fecchio R (2018). Morning vs evening aerobic training effects on blood pressure in treated hypertension. Med Sci Sports Exerc.

[44] Gabriel BM, Zierath JR (2019). Circadian rhythms and exercise—re-setting the clock in metabolic disease. Nat Rev Endocrinol.

[45] Holtermann A, Krause N, Van Der Beek AJ, Straker L (2018). The physical activity paradox: six reasons why occupational physical activity (OPA) does not confer the cardiovascular health benefits that leisure time physical activity does. Br J Sports Med.

[46] Thomas JM, Kern PA, Bush HM (2020). Circadian rhythm phase shifts caused by timed exercise vary with chronotype. JCI Insight.

[47] Yu JH, Yun C-H, Ahn JH (2015). Evening chronotype is associated with metabolic disorders and body composition in middle-aged adults. J Clin Endocrinol Metab.

