# Supplementary material for: Timing of physical activity in relation to liver fat content and insulin resistance
Source: Diabetologia. 2022 Nov 1;66(3):461–71. doi: 10.1007/s00125-022-05813-3 (PMC9892088; doi:10.1007/s00125-022-05813-3)
Supplement: Supplementary file 1 — (PDF 491 kb) [file 125_2022_5813_MOESM1_ESM.pdf]

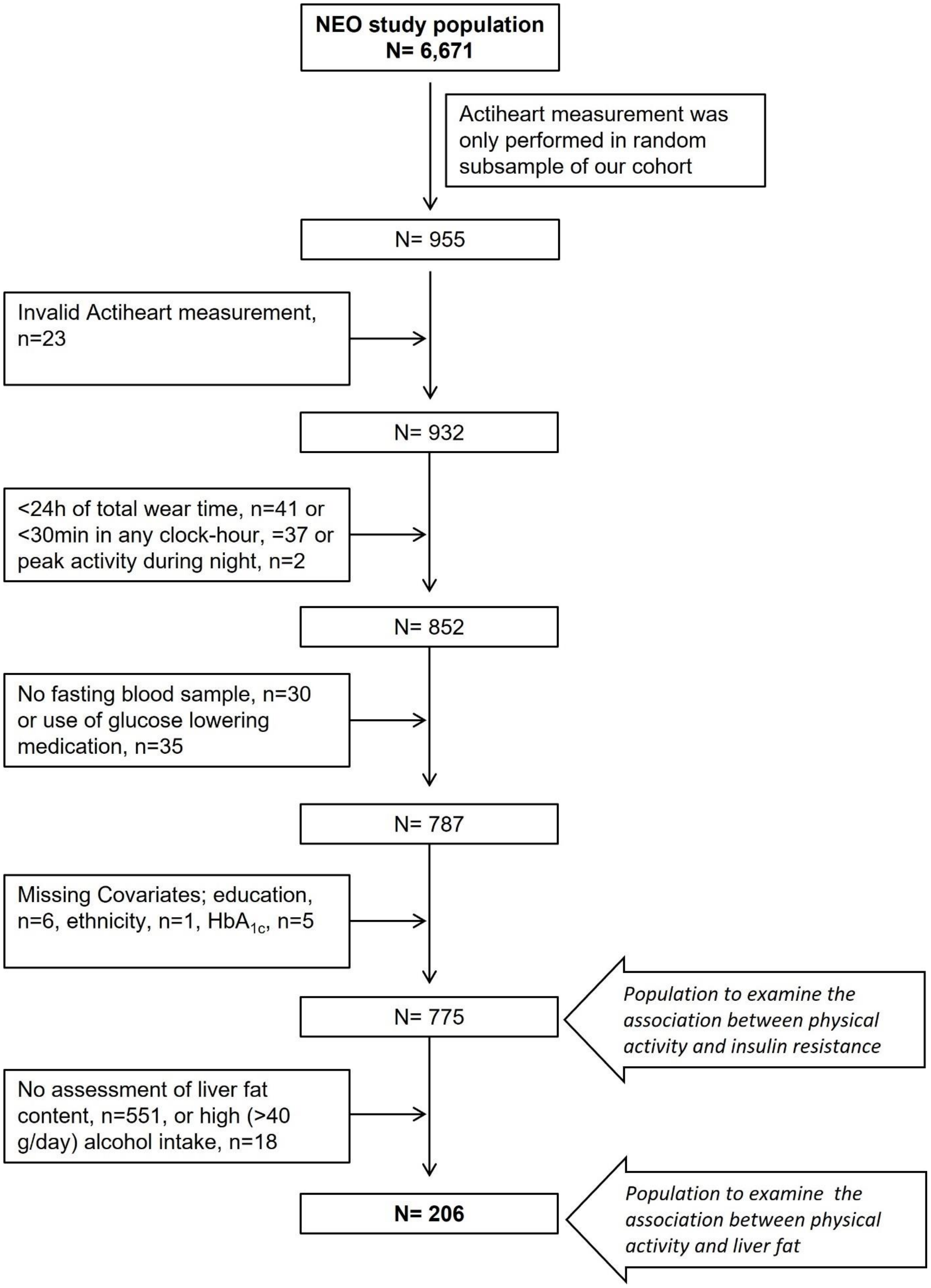

ESM Figure 1: flowchart to illustrate selection of study population

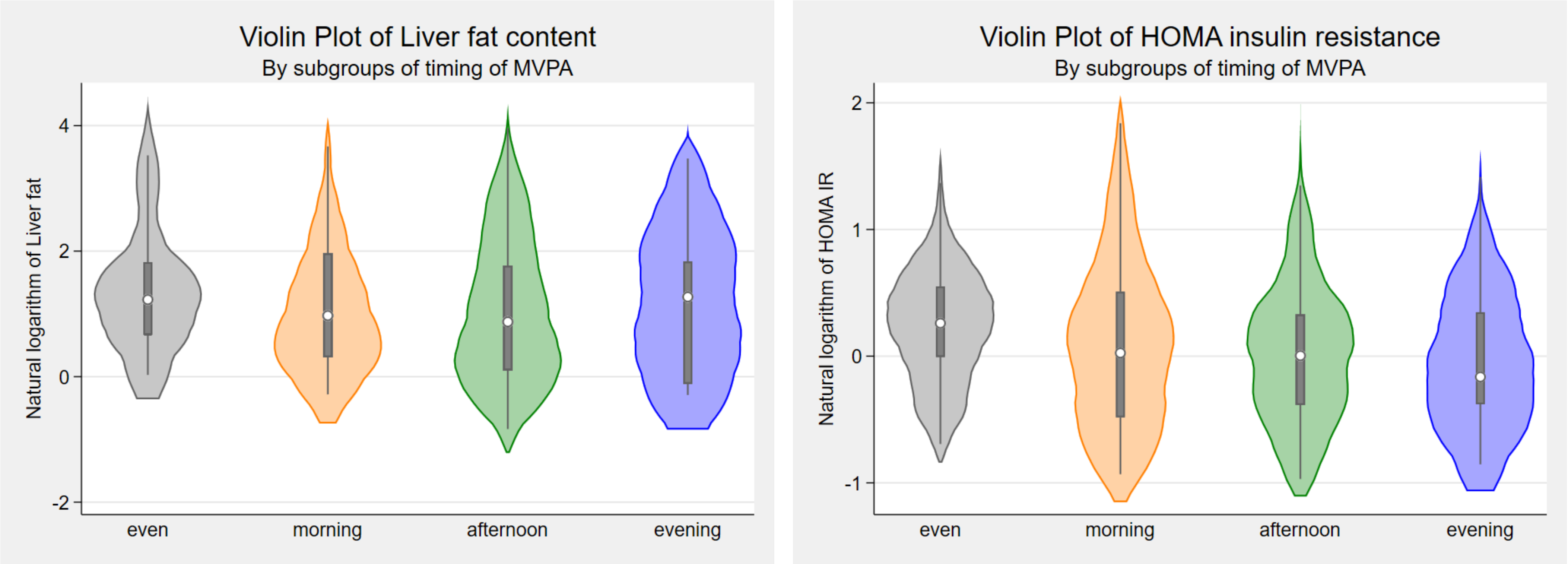

ESM Figure 2: Crude difference in liver fat content (left) and insulin resistance (right) between subgroups based on timing of MVPA. Due to skewed distribution, Y-axis is in natural log scale. Results are based on analyses weighted toward the BMI distribution of the general population. Associations with insulin resistance were based on the total study population (n=775), those with liver fat content on n=206 with liver fat content measured by ^{1}H-. Participants with alcohol consumption >=40 g per day were excluded from the analyses with liver fat content (n=18).

ESM Table 1 relative changes in insulin sensitivity, fasting levels of glucose, insulin, and HbA_{1c} for sedentary time, number of sedentary breaks and different levels of physical activity intensity

		Relative change (95% CI)			
		**Matsuda ISI**	**Fasting glucose**	**Fasting insulin**	**HbA1c**
Sedentary time (ST, h/day)	Model 1	0.97 (0.95-1.00)	1.00 (1.00-1.01)	1.02 (0.99-1.04)	1.00 (1.00-1.00)
	Model 2	0.98 (0.94-1.00)	1.00 (1.00-1.01)	1.02 (0.99-1.04)	1.00 (1.00-1.00)
	Model 3	0.99 (0.97-1.01)	1.00 (1.00-1.01)	1.01 (0.98-1.03)	1.00 (1.00-1.00)
Sedentary breaks acceleration (10/day)	Model 1	1.03 (0.99-1.07)	1.00 (0.99-1.01)	0.97 (0.93-1.01)	1.00 (1.00-1.00)
	Model 2	1.03 (0.99-1.08)	1.00 (0.99-1.01)	0.97 (0.93-1.01)	1.00 (1.00-1.00)
	Model 3	1.01 (0.98-1.05)	1.00 (1.00-1.01)	0.98 (0.95-1.02)	1.00 (1.00-1.01)
	Model 3 + ST and MVPA	1.02 (0.98-1.06)	1.00 (1.00-1.01)	0.98 (0.94-1.02)	1.00 (1.00-1.00)
Total PAEE (10 kj/kg/day)	Model 1	1.06 (1.02-1.10)	0.99 (0.99-1.00)	0.96 (0.93-0.98)	1.00 (1.00-1.00)
	Model 2	1.06 (1.02-1.09)	0.99 (0.99-1.00)	0.96 (0.93-0.99)	1.00 (1.00-1.00)
	Model 3	1.03 (1.00-1.07)	1.00 (0.99-1.00)	0.98 (0.95-1.00)	1.00 (1.00-1.00)
Light physical activity (LPA, h/day)	Model 1	1.02 (0.98-1.06)	1.00 (0.99-1.01)	0.99 (0.96-1.02)	1.00 (1.00-1.01)
	Model 2	1.02 (0.98-1.06)	1.00 (0.99-1.01)	0.99 (0.96-1.02)	1.00 (1.00-1.01)
	Model 3	1.00 (0.97-1.04)	1.00 (0.99-1.01)	1.00 (0.97-1.03)	1.00 (1.00-1.01)
Moderate-to-vigorous physical activity (MVPA, h/day)	Model 1	1.12 (1.05-1.20)	0.98 (0.97-1.00)	0.92 (0.87-0.97)	1.00 (0.99-1.00)
	Model 2	1.11 (1.04-1.18)	0.99 (0.97-1.00)	0.92 (0.87-0.97)	1.00 (0.99-1.00)
	Model 3	1.08 (1.02-1.15)	0.99 (0.98-1.01)	0.95 (0.90-1.00)	1.00 (0.99-1.01)
MVPA in 5-minute bouts (h/day)	Model 1	1.20 (1.11-1.30)	0.98 (0.96-1.00)	0.87 (0.81-0.93)	1.00 (0.99-1.00)
	Model 2	1.18 (1.10-1.27)	0.98 (0.96-1.00)	0.87 (0.81-0.94)	1.00 (0.99-1.00)
	Model 3	1.14 (1.06-1.22)	0.99 (0.97-1.01)	0.92 (0.86-0.98)	1.00 (0.99-1.01)

Results are based on analyses weighted toward the BMI distribution of the general population and were derived from β coefficients with 95% CIs from linear regression analyses and expressed as a relative change. Such a ratio, e.g 0.8, can be interpreted as 0.8 fold liver fat content for each hour of MVPA per day, which would reflect a decrease in liver fat content from, for example, 5% to 4%. Further, these relative changes were expressed as percentage change in the text: (exp(beta) -1)*100 if beta >0 and: -(1/exp(-beta) -1)*100 if beta <0 with 95% confidence intervals, e.g. a relative change of 0.80 (0.67, 0.96) corresponds with -25% (-49%,-4%) percentage change.
Model 1: associations were adjusted for age, sex, educational background and ethnicity
Model 2: as for model 1, additionally adjusted for alcohol consumption, smoking and the Dutch healthy diet index
Model 3: as for model 2, additionally adjusted for total body fat
.

ESM Table 2 relative changes in liver fat content, insulin resistance, insulin sensitivity, fasting levels of glucose, insulin, and HbA_{1c} between subgroups based on timing of **MVPA** and subgroups based on timing of **MVPA in 5-minute bouts**

		relative change (95% CI)					
		**Liver fat content**	**Insulin resistance**	**Matsuda ISI**	**Fasting glucose**	**Fasting insulin**	**HbA1c**
Most MVPA throughout the day	Even (12%)	1	1	1	1	1	1
	Morning (16%)	1.13 (0.69-1.83)	0.97 (0.80-1.16)	0.93 (0.78-1.12)	1.03 (0.99-1.07)	0.96 (0.80-1.16)	1.01 (1.00-1.03)
	Afternoon (63%)	1.13 (0.75-1.69)	0.85 (0.75-0.98)	1.09 (0.95-1.24)	1.01 (0.99-1.04)	0.85 (0.74-0.98)	1.01 (1.00-1.02)
	Evening (7%)	1.08 (0.56-2.07)	0.80 (0.67-0.96)	1.06 (0.87-1.29)	1.03 (0.98-1.08)	0.79 (0.66-0.95)	1.03 (1.00-1.05)
Most MVPA in 5-min bouts throughout the day	Even (11%)	1	1	1	1	1	1
	Morning 19%)	0.87 (0.53-1.41)	0.93 (0.76-1.14)	1.06 (0.85-1.32)	1.00 (0.95-1.05)	0.93 (0.76-1.14)	1.01 (0.99-1.03)
	Afternoon (54%)	1.10 (0.70-1.71)	0.84 (0.71-1.00)	1.17 (0.96-1.44)	1.01 (0.97-1.06)	0.84 (0.71-1.00)	1.02 (1.00-1.03)
	Evening (16%)	1.02 (0.60-1.72)	0.86 (0.70-1.05)	1.12 (0.89-1.41)	1.02 (0.97-1.08)	0.85 (0.70-1.04)	1.02 (1.00-1.05)

Associations were adjusted for age, sex, educational background, ethnicity, alcohol consumption, smoking, Dutch healthy diet index, total body fat percentage, and total MVPA. Results are based on analyses weighted toward the BMI distribution of the general population and were derived from β coefficients with 95% CIs from linear regression analyses and expressed as a relative change. Such a ratio, e.g. 0.8, can be interpreted as 0.8 fold liver fat content compared with an even distribution of MVPA, which would reflect a decrease in liver fat content from, for example, 5% to 4%. Further, these relative changes were expressed as percentage change in the text: (exp(beta) -1)*100 if beta >0 and: -(1/exp(-beta) -1)*100 if beta <0 with 95% confidence intervals, e.g. a relative change of 0.80 (0.67, 0.96) corresponds with -25% (-49%,-4%) percentage change.

ESM Table 3 relative changes in liver fat content, insulin resistance, insulin sensitivity, fasting levels of glucose, insulin, and HbA_{1c} between subgroups based on where most of total physical activity energy expenditure (**PAEE**) or light physical activity (**LPA**) took place

		Relative change (95% CI)					
		**Liver fat content**	**Insulin resistance**	**Matsuda ISI**	**Fasting glucose**	**Fasting insulin**	**HbA1c**
Most PAEE throughout the day	Even (21%)	1	1	1	1	1	1
	Morning (11%)	0.77 (0.48-1.24)	0.84 (0.70-1.00)	1.12 (0.94-1.33)	0.99 (0.96-1.03)	0.84 (0.70-0.99)	1.01 (1.00-1.03)
	Afternoon (62%)	0.88 (0.59-1.33)	0.88 (0.78-0.99)	1.10 (0.98-1.25)	1.01 (0.98-1.04)	0.88 (0.78-0.99)	1.01 (1.00-1.03)
	Evening (6%)	1.19 (0.60-2.37)	0.88 (0.71-1.08)	1.03 (0.80-1.31)	1.05 (0.99-1.11)	0.86 (0.70-1.06)	1.03 (1.00-1.07)
Most LPA throughout the day	Even (28%)	1	1	1	1	1	1
	Morning (9%)	0.92 (0.53-1.62)	0.90 (0.75-1.08)	1.16 (0.97-1.41)	0.98 (0.94-1.02)	0.90 (0.75-1.08)	0.99 (0.98-1.01)
	Afternoon (59%)	1.39 (1.01-1.90)	1.02 (0.92-1.13)	0.98 (0.88-1.10)	0.99 (0.97-1.02)	1.02 (0.92-1.13)	1.00 (0.99-1.01)
	Evening (5%)	1.29 (0.71-2.36)	1.02 (0.80-1.32)	0.97 (0.76-1.25)	1.02 (0.98-1.06)	1.02 (0.79-1.31)	1.01 (0.98-1.04)

Associations were adjusted for age, sex, educational background, ethnicity, alcohol consumption, smoking, Dutch healthy diet index, total body fat percentage, and total PAEE, or LPA , respectively. Results are based on analyses weighted toward the BMI distribution of the general population and were derived from β coefficients with 95% CIs from linear regression analyses and expressed as a relative change. Such a ratio, e.g. 0.8, can be interpreted as 0.8 fold liver fat content for each hour of MVPA per day, which would reflect a decrease in liver fat content from, for example, 5% to 4%. Further, these relative changes were expressed as percentage change in the text: (exp(beta) -1)*100 if beta >0 and: -(1/exp(-beta) -1)*100 if beta <0 with 95% confidence intervals, e.g. a relative change of 0.80 (0.67, 0.96) corresponds with -25% (-49%,-4%) percentage change.
